# Variance in heat tolerance in bumble bees correlates with species geographic range and is associated with several environmental and biological factors

**DOI:** 10.1002/ece3.10730

**Published:** 2023-11-28

**Authors:** Cody Feuerborn, Gabriela Quinlan, Rachael Shippee, Tori L. Strausser, Tatiana Terranova, Christina M. Grozinger, Heather M. Hines

**Affiliations:** ^1^ Department of Biology Pennsylvania State University University Park Pennsylvania USA; ^2^ Department of Entomology, Center for Pollinator Research, Huck Institutes of the Life Sciences Pennsylvania State University University Park, State College Pennsylvania USA; ^3^ Department of Biology Utah State University Logan Utah USA; ^4^ Department of Molecular Genetics and Microbiology Duke University Medical Center Durham North Carolina USA

**Keywords:** bumble bee, climate change, conservation, physiology, thermal tolerance

## Abstract

Globally, insects have been impacted by climate change, with bumble bees in particular showing range shifts and declining species diversity with global warming. This suggests heat tolerance is a likely factor limiting the distribution and success of these bees. Studies have shown high intraspecific variance in bumble bee thermal tolerance, suggesting biological and environmental factors may be impacting heat resilience. Understanding these factors is important for assessing vulnerability and finding environmental solutions to mitigate effects of climate change. In this study, we assess whether geographic range variation in bumble bees in the eastern United States is associated with heat tolerance and further dissect which other biological and environmental factors explain variation in heat sensitivity in these bees. We examine heat tolerance by caste, sex, and rearing condition (wild/lab) across six eastern US bumble bee species, and assess the role of age, reproductive status, body size, and interactive effects of humidity and temperature on thermal tolerance in *Bombus impatiens*. We found marked differences in heat tolerance by species that correlate with each species' latitudinal range, habitat, and climatic niche, and we found significant variation in thermal sensitivity by caste and sex. Queens had considerably lower heat tolerance than workers and males, with greater tolerance when queens would first be leaving their natal nest, and lower tolerance after ovary activation. Wild bees tended to have higher heat tolerance than lab reared bees, and body size was associated with heat tolerance only in wild‐caught foragers. Humidity showed a strong interaction with heat effects, pointing to the need to regulate relative humidity in thermal assays and consider its role in nature. Altogether, we found most tested biological conditions impact thermal tolerance and highlight the stages of these bees that will be most sensitive to future climate change.

## Graphical abstract

This research assesses the biological and environmental factors that contribute to heat tolerance in bumble bees in the eastern Nearctic. Heat tolerance varied by species as expected given their distribution and habitat preferences, queens were found to be more heat sensitive than workers and males, and age, physiological state, rearing conditions, body size, and humidity were all found to influence heat tolerance in these bees.

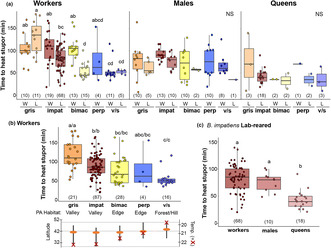

## 1 INTRODUCTION

Across much of the northern hemisphere and South America, where the ~270 bumble bee (Hymenoptera: Apidae: *Bombus* spp.) species are native, numerous flowering agricultural crops and wild flowering species have evolved to be heavily dependent on bumble bees for their pollination (Goulson, 2010). These services, however, are under threat given documented declines in these bees over the last century (Cameron et al., 2011; Goulson et al., 2008; Soroye et al., 2020). In Europe and North America, 26% of bumble bee species now are listed as threatened or endangered (Cameron & Sadd, 2020), and bumble bee species richness has declined 30% in the Northeastern United States since the late 1800s (Bartomeus et al., 2018). While there are many stressors impacting bee populations (e.g., pathogens, habitat loss, and pesticides), climate change is a major factor linked to range shifts and biodiversity loss in bees and other pollinators (Soroye et al., 2020; Vasiliev & Greenwood, 2021), with several studies in bumble bees exemplifying these effects.

Bumble bees are particularly vulnerable to increasing temperatures associated with climate change. Hamblin et al. (2017) found bumble bees to be most susceptible to heat among 15 tested bee species in North Carolina, U.S.A., and Pardee et al. (2022) found that bumble bees were less abundant during warmer years compared to 28 other bee genera in a montane region in Colorado, U.S.A. Although bumble bees could benefit from some warming as it might reduce their expenditures toward maintaining endothermy (Jackson et al., 2020), bumble bees have been found to decrease foraging and their colonies to have decreased growth with high heat (Gérard et al., 2022; Hemberger et al., 2023; Vanderplanck et al., 2019). Effects of climate change in bumble bees have been well documented using large‐scale geographic and climate modeling approaches. In Europe and North America, climate has been found to be a better predictor than habitat in explaining declines, and regions with greater increase in temperature have experienced greater declines in bumble bee diversity (Soroye et al., 2020; although see Guzman et al., 2021). Bumble bee species have also experienced range contraction in response to climate warming, with southern ranges receding northward without a corresponding shift at the northern edges of their ranges (Kerr et al., 2015). Bumble bees may also be shifting altitudinally with climate, potentially creating isolated populations that are vulnerable to extirpation (Kerr et al., 2015; Pyke et al., 2016). Forecasting models taking into account climatic niches and dispersal abilities of bumble bees predict that many species will fail to disperse into new climatically suitable conditions with projected climate change, leading to declining populations (Sirois‐Delisle & Kerr, 2018).

Not all bumble bee species have declined equally in the face of recent climate and land use changes (Cameron et al., 2011; Colla et al., 2012; Grixti et al., 2009). Determining the factors that make species resilient requires a better understanding of the factors that limit species distributions. Bumble bees are cold‐adapted with the highest species diversity in cool, temperate‐boreal, and montane habitats, although a few species are endemic to deserts and tropical lowlands (Hines, 2008; Williams et al., 2014). Recent studies point to cold tolerance being a limiting factor on altitudinal and geographic distribution, and cold tolerance is also likely under selection (Jackson et al., 2020; Keaveny et al., 2019, 2023; Maebe et al., 2021; Oyen et al., 2016; Pimsler et al., 2020). However, the global distributions of bumble bee species appear to be further influenced by species‐level variation in heat tolerance (Table 1): Martinet, Dellicour, et al. (2021) performed an analysis of thermal tolerance on males of 39 bumble bee species across Europe and North America and found interspecific variation in heat stress tolerance that matches species distributions. Arctic and boreal species had lower resistance to hyperthermic stress than temperate and Mediterranean species. The high heat tolerance in the most southern distributed Mediterranean species *Bombus terrestris* (Linnaeus) is particularly striking given that this species is generally expanding its range. Oyen and Dillon (2018) likewise found that the lower altitude Rocky Mountain species *Bombus huntii* Greene had higher heat tolerance than *Bombus sylvicola* Kirby, which inhabits higher altitudes. Gonzalez et al. (2022) applied thermal tolerance data from other studies and noted a relationship between heat tolerance and several climatic variables in five North American bumble bee species. Differential heat tolerance in bumble bees may be conferred through differences in molecular heat shock response (Blasco‐Lavilla et al., 2021; Kuo et al., 2023; Pimsler et al., 2020), interspecific differences in heat shunting by means of counter‐current exchange between the thorax and abdomen (cf. Heinrich, 1976; Heinrich & Vogt, 1993), or through other physiological adaptations such as shifting metabolism or potential for evaporative cooling (Johnson et al., 2023).

**TABLE 1:** Summary of the literature on bumble bee heat tolerance for the parameters examined here. Provided are the parameters studied, whether or not there was an effect (“Eff?”) on heat tolerance, the bumble bee species studied, how the parameters relate to heat tolerance, and the citation of the study.

Parameters	Eff.?	*Bombus* spp.	Relationship	Reference
Species	Yes	39 spp. From three continents	Higher THS in species from warmer habitats	Martinet, Dellicour, et al. (2021)
	Yes	*huntii*, *bifarius*, and *sylvicola*	CT_max_ higher in *B. huntii* than *B. sylvicola*	Oyen et al. (2016)
	No	*hortulanus*, *funebris*, *pauloensis*, and *rubicundus*	CT_max_ did not differ among these high‐elevation Colombian species	Gonzalez et al. (2022)
	Yes	Five spp. from Europe	THS was higher in Boreo‐alpine than arctic species	Martinet et al. (2015)
	Yes	10 spp. from Belgium	THS was higher in *B. lucorum* and *B. terrestris* than other species	Zambra et al. (2020)
	Yes	Five N. American spp.	CT_max_ relates to several climate variables	Gonzalez et al. (2022)
Latitude	No	*vosnesenskii*	CT_max_ invariant from queens originating at different latitudes	Pimsler et al. (2020)
Altitude	Yes	*huntii*, *bifarius*, and *sylvicola*	Low altitude species had higher CT_max_	Oyen et al. (2016)
	No	*hortulanus*, *funebris*, *pauloensis*, and *rubicundus*	CT_max_ did not differ between low‐ and high‐elevation species	Gonzalez et al. (2022)
Body size/mass	No	*impatiens*	CT_max_ not different by body mass in laboratory‐reared workers	Oyen and Dillon (2018)
	Yes	*huntii*, *bifarius*, and *sylvicola*	CT_max_ increased with body mass in *B. huntii*	Oyen et al. (2016)
	No	*B. terrestris* (three subspp.)	CT_max_ not correlated with body size in laboratory‐reared workers or queens	Maebe et al. (2021)
	No	*hortulanus*, *funebris*, *pauloensis*, and *rubicundus*	CT_max_ not correlated with ITD comparing all sexes and species	Gonzalez et al. (2022)
	Yes	39 spp. From three continents	Body mass weakly explained variance in THS in wild males	Martinet, Dellicour, et al. (2021)
	No	10 spp. from Belgium	THS was not related to body size of wild bees (ITD)	Zambra et al. (2020)
	No	*B. terrestris audax*	CT_max_ did not relate to body mass in laboratory‐reared workers	Sepúlveda and Goulson (2023)
Caste/sex	No	*terrestris* (three subspp.)	CT_max_ not correlated with caste (workers vs. queens)	Maebe et al. (2021)
	No	*huntii*, *bifarius*, and *sylvicola*	CT_max_ did not differ between males and workers	Oyen et al. (2016)
	No	*hortulanus*, *funebris*, *pauloensis*, and *rubicundus*	CT_max_ did not differ between males and workers	Gonzalez et al. (2022)
Age	Yes	*impatiens*	CT_max_ was lower in 4‐day‐old than 3‐ and 7‐day‐old worker bees	Oyen and Dillon (2018)
Wild versus laboratory	Yes	*pauloensis*	CT_max_ was higher and thermal breadth greater for laboratory‐reared bees	Gonzalez et al. (2022)

The susceptibility of bumble bees to climate is likely to vary not only across species but by life history and condition within species. Prior studies examining species heat tolerance have shown high variance across individuals within species (Martinet, Dellicour, et al., 2021; Oyen & Dillon, 2018), suggesting that other biological factors interplay with thermal physiology to impact tolerance. Bumble bees, as social species, exhibit variation across individuals in physiology and behavior depending on caste and stage of their life cycle (Amsalem, Grozinger, et al., 2015). Queen bumble bees, for example, are known to shift their nutritional reserves in preparation for diapause and upon ovary activation (Amsalem, Galbraith, et al., 2015; Treanore & Amsalem, 2022), and workers in late or queen‐less colonies can also lay eggs (Goulson, 2010). There may also be selection for bumble bees in different roles to have different heat tolerances based on differences in thermal exposure. Bumble bees typically nest underground where they tend to be buffered from the elements, although some species tend toward surface nesting (Liczner & Colla, 2020). Males are exposed to environmental conditions more than workers, as workers can escape the heat in nests (Heinrich, 2004). Among workers, foragers are more exposed to heat stress than nurses, which usually reside only in the nest. Queens in general receive little exposure to heat, as they are typically produced in late summer where they initially feed in natal nests, subsequently receiving the most exposure when they leave the nest to mate (~1–2 weeks postemergence; Röseler & van Honk, 1990), after which they dig themselves a hibernaculum underground for overwintering. They will emerge again in the cool of spring to forage for provisions for their nest, thereafter remaining protected in underground colonies (Gardner et al., 2007).

There have been a few studies examining how thermal heat tolerance in bumble bees varies with sex and caste (Table 1). Maebe et al. (2021) found differences between *B. terrestris* workers and queens in critical minimum temperatures (CT_min_) but saw no difference in critical maximum temperatures (CT_max_) between castes. Oyen et al. (2016) found no difference in CT_max_ between males and workers within three Rocky Mountain species (*B. huntii*, *B. bifarius* Cresson, and *B. sylvicola*) and Gonzalez et al. (2022) found no role of sex in thermal tolerance in Colombian bumble bees. The role of reproductive state has yet to be examined.

Age‐related variation in thermal tolerance has been documented in a number of holometabolous insects with many species showing a decline in tolerance with age (Bowler & Terblanche, 2008). Bumble bee workers tend to live from 2 to 6 weeks and can switch roles from in‐nest and nursing tasks to foraging as they age (Tobback et al., 2011). A study examining the critical thermal limits of *Bombus impatiens* Cresson at 3, 4, and 7 days, found 3‐day‐old and 7‐day‐old workers are more heat resistant than 4‐day‐old workers (Oyen & Dillon, 2018). The study, however, did not test the effect of older ages (>1 week) on thermal tolerance.

Bumble bees are facultative endotherms and can maintain constant body temperatures through a wide range of air temperatures (Church, 1960; Goulson, 2010; Heinrich, 2004). Nevertheless, by Bergmann's rule, the small surface to volume (S/V) ratio of larger bees should reduce their thermoregulatory capabilities for dissipating heat (Heinrich, 2004), while increasing their ability to retain heat in cold conditions. Given evolutionary tradeoffs in body size in insects, however, Bergmann's rule may not always apply (Lozier et al., 2021; Shelomi, 2012). Prior work found that CT_max_ increased with mass in reared workers of *B. huntii* (Oyen et al., 2016), but not in wild workers of *B. bifarius*, *B. sylvicola* (Oyen et al., 2016), *B. impatiens* (Oyen & Dillon, 2018), or *B. terrestris* (Maebe et al., 2021). Martinet, Dellicour, et al. (2021) found only weak explanatory power of body mass on time to heat stupor at 40°C among males of 39 bumble bee species. Recent studies have found a decrease in body size in workers in the last 100+ years in certain bumble bees (Nooten & Rehan, 2020) and other bee populations (Oliveira et al., 2016). Although developmental plasticity could be involved in this, it is possible that climate change may be implicated via natural selection if larger bees are more negatively impacted by heat. More evidence is needed to understand if body size indeed plays a role in heat stress tolerance and long‐term shifts in body size in bumble bees.

Nutrition may also impact thermal tolerance (Vanderplanck et al., 2019) and, if so, this can both explain natural variance in heat tolerance and present a solution to mediate effects of thermal stress. There have been mixed results on the effects of starvation on thermal tolerance with some studies finding a role (Blasco‐Lavilla et al., 2021; Quinlan et al., 2023) and another seeing no correlation (Oyen & Dillon, 2018).

In this study, we sought to better understand which factors contribute to heat stress and resilience in bumble bees. In particular, we sought to (i) better understand what biological factors (e.g., age, caste, sex, or rearing conditions) contribute to variance in thermal tolerance, (ii) determine points of vulnerability within the life cycle, (iii) better understand whether heat tolerance may be a factor limiting distribution and niche, and (iv) understand whether heat tolerance may impact success of different species in the face of climate change. Toward these goals, we examine heat stress tolerance variation across six species native to the Northeastern United States: *B. impatiens*, *Bombus bimaculatus* Cresson, *Bombus griseocollis* (De Geer), *Bombus perplexus* Cresson, *Bombus vagans* Smith, and *Bombus sandersoni* Franklin. These species have different but overlapping distributions and habitat preferences (Gratton et al., 2023; Williams et al., 2014) and vary in their abundance in our study region in Pennsylvania. In particular, *B. impatiens* is increasing in abundance, *B. bimaculatus* and *B. griseocollis* are stable or increasing, and *B. perplexus*, *B. vagans*, and *B. sandersoni* are more rare and potentially decreasing (Colla et al., 2012; Grixti et al., 2009; Jacobson et al., 2018). In terms of habitat, *B. impatiens* and *B. griseocollis* are more abundant in open valleys, *B. sandersoni* and *B. vagans* in the ridges, and *B. perplexus* and *B. bimaculatus* in ecotonal regions (Gratton et al., 2023; Williams et al., 2014). To understand how aspects of life history within species impact thermal tolerance and vulnerability, we examine the role of sex and caste across species, and of age, body size, and reproductive state of queens on thermal tolerance in *B. impatiens*. As an indirect test of potential impacts of environmental factors like nutrition, we compare wild foraging workers to laboratory‐reared bees. Finally, we examine the impact of humidity levels on resulting heat stress tolerance. Humidity and temperature interactions may be important as high humidity can reduce the potential for evaporative cooling in insects as it reduces the hygric gradient between the bee and environment (Church, 1960; Prange, 1996). While evaporative cooling is thought to not be a major factor in most bees (Heinrich, 2004; Johnson et al., 2023), in bumble bees high humidity has been found to reduce cooling during flight (Church, 1960). Altogether, our data reveal that many biological factors impact thermal tolerance in bumble bees, providing a baseline for understanding points of vulnerability and for guiding solutions to better manage bumble bees under climate change.

## 2 METHODS

### 2.1 Bee specimen sources

**FIGURE 1:**
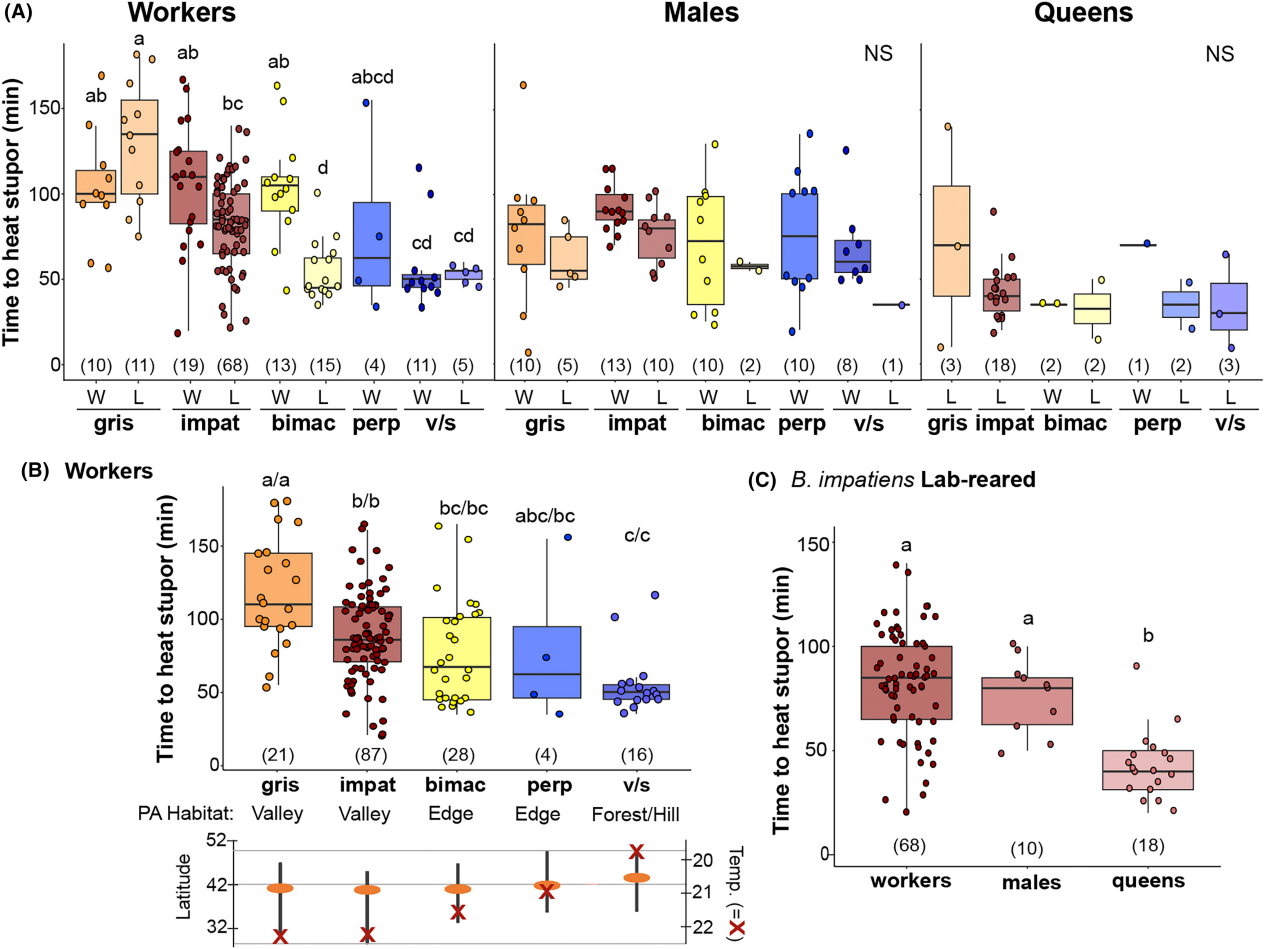
Thermal tolerances (time to heat stupor, THS) of different bumble bee species, castes, and in wild versus laboratory‐reared bees. (A) Interspecific differences in THS across all variables, highlighting differences by species, sex/caste, and between wild (W) versus laboratory‐reared (L) bees for each caste‐species combination. For ease of viewing, significance letters in 1a represent pairwise differences among species/origin (Tukey HSD) within each caste. (B) Interspecific differences among workers (wild and laboratory‐reared bees pooled) highlighting significance values and how THS of species directly relates to their latitudinal averages of GBIF records, mean temperature (°C) across this range for June, July, and August (indicated with Xs), and habitat preferences across a forested hill to open and developed valley landscape in Pennsylvania (Gratton et al., 2023). Significance letters on the left of the slash represent pairwise differences (Tukey HSD) between workers of different species and letters on the right of the slash represent pairwise differences (Tukey HSD) between species from the full model, which includes workers, males, and queens in the analysis. (C) Sex/caste differences in laboratory‐reared *Bombus impatiens*, which had the best sample sizes to observe caste differences, and their significance. Sample sizes for each condition are indicated in parentheses. bimac, *Bombus bimaculatus*; gris, *Bombus griseocollis*; imp, *Bombus impatiens*; perp, *Bombus perplexus*; v/s, *Bombus vagans*/*sandersoni*.

Bees for our thermal trials were sourced either directly from the wild (collected as foraging queens, workers, or males), drawn from laboratory‐reared colonies founded by wild‐caught queens, or from commercially purchased colonies (thus from laboratory‐reared queens). While we sought to balance replicates in the design as much as possible with our sampling effort, sample size of field‐collected bees was somewhat uneven across groups due to limited field availability. A total of 379 bees were tested for these assays with sample sizes and species information contained in Table A1, with a more detailed table in Scholarsphere (https://doi.org/10.26207/gqy8‐e215), and apparent in Figure 1A.

#### 2.1.1 Wild bees

**FIGURE A1:**
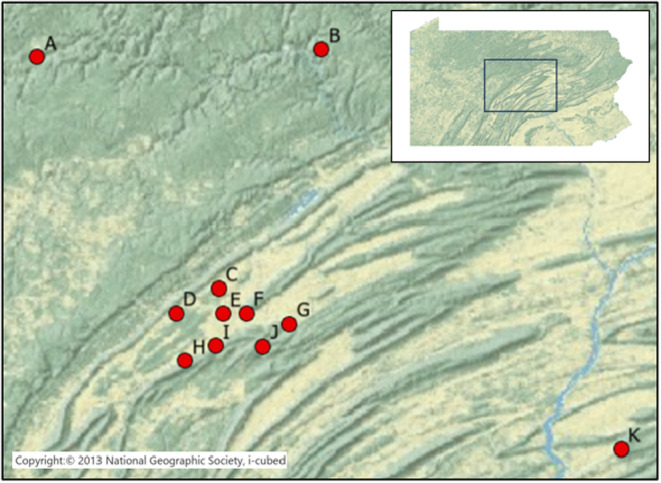
Map of collection sites for wild bees used in THS assays. Elk Country Visitor Center (a), Hyner View State Park (b), Bernel Road Park (c), Circleville Park (d), Penn State–University Park Campus (e), Tom Tudek Memorial Park (f), Colyer Lake (g), The Russell E. Larson Agricultural Research Center at Rock Springs (h), Pine Grove Mills (i), Bear Meadows Natural Area (j), and Weiser State Forest (k).

A bee is defined as wild if thermal tolerance was tested within 24 h of its collection from nature. The Northeast U.S., and Pennsylvania in particular, is considered to have a humid continental climate (“Pennsylvania State Climatologist”, n.d.), characterized by widely varying temperatures between summer and winter. Wild‐caught bees for our study were collected from central Pennsylvania's Ridge and Valley Province and Allegheny Plateau which have greater temperature extremes than the Southeastern Coastal Plain and Piedmont Plateau (“Pennsylvania State Climatologist”, n.d.). Bumble bee field collections were conducted from June 29th to November 8th, 2022, and from July 25th to July 26th, 2023, across 11 sites in Pennsylvania (Figure A1), each within a 1.5‐h drive from the research laboratory at Penn State's University Park campus. Bees were collected by hand netting in an opportunistic manner, mostly while foraging on flowers, and transported in vials from the field at ~7°C in a cooler containing ice packs to slow down their activity during transport. Once in the laboratory, bees were transferred into plastic cages, provisioned ad libitum with pollen patties (pollen collected from honey bees; sourced from Swarmbustin’ Honey Bee Farm, Westgrove, PA) and artificial nectar used for laboratory colonies (see below), and placed in a walk‐in environmental chamber set to 28°C, ~65% relative humidity (RH) for 12–16 h prior to trials (cf. Martinet et al., 2015).

#### 2.1.2 Laboratory‐reared bees from wild queens

We identified bees as laboratory‐reared if they were drawn for thermal tolerance experiments from lab reared colonies. For laboratory‐reared bees from wild queens, spring foundresses of each of the species/species‐groups were collected from the field while either foraging or nest searching (April 24th–May 17th, 2022) and used to rear bumble bee colonies in the laboratory, resulting in five *B. impatiens*, four *B. griseocollis*, four *B. bimaculatus*, two *B. perplexus*, and three *B. vagans*/*sandersoni* source colonies. For this, wild queens were chilled and brought back to the laboratory, kept in an incubator at 28°C, ~65% RH, and provisioned with a bee pollen patty blended with Biogluc proprietary nectar (Biobest, Canton, MI) or a laboratory‐made sugar solution ad libitum throughout the colony development. The laboratory‐made solution was composed of 50% sucrose and 50% invert sugar with amino acid supplement (Amino‐B Booster; Honey‐B‐Healthy Inc., Cumberland, MD) to provide bees with essential amino acids.

#### 2.1.3 Laboratory‐reared bees from commercial colonies

For some of our experiments, we assayed workers, males, and newly emerged queens (we use the term “queen” here to include both mated or unmated and those that have or have not started a nest) produced from commercial, research‐grade *B. impatiens* colonies (sourced from Biobest, Canton, MI). Commercial colonies were kept under the same conditions as laboratory‐reared wild queen colonies.

### 2.2 Time to heat stupor assays: Cross‐species comparisons

#### 2.2.1 Time to heat stupor protocol

We used time to heat stupor (THS) as a measure of thermal stress across all bees (Martinet, Dellicour, et al., 2021). Symptoms of heat stupor include a loss of neuromuscular function, which is characterized by uncoordinated movements, the inability right itself when flipped on its back, twitching in the extremities (e.g., tarsi), and heat coma (cf. Martinet, Dellicour, et al., 2021). We used THS instead of CT_max_ (critical maximum temperature, the temperature at which bees reach heat stupor following an incremental temperature ramping rate from ambient temperature; Oyen & Dillon, 2018), as we sought to test thermal endurance without the confounding factor of ramping rate. We felt THS would be more akin to the temporal response upon entering a hot environment from a cool nest. Moreover, THS has been useful for discriminating bumble bee species‐specific tolerances in the past (Martinet, Dellicour, et al., 2021; Zambra et al., 2020). Static (THS) and dynamic (CT_max_) assays of heat tolerance have been found to be comparable and thus reliable methods of assessing heat tolerance in *Drosophila* (Jørgensen et al., 2019) and thus these methods should yield similar results in bumble bees, however, we recognize that the method applied will likely affect magnitudes and variance of response. Trials were limited to 3 h to avoid confounding effects of starvation on THS. We thus consider bees reaching 3 h as “heat resistant.” In our preliminary THS trials, we used 40°C as our critical temperature. This temperature has been used in previous studies to induce heat stupor across bumble bee species (Martinet, Dellicour, et al., 2021; Zambra et al., 2020) and is above the threshold temperature commonly used to define a heat wave (Xu et al., 2016). However, most bees in our preliminary study were reaching 3 h without entering heat stupor, so we decided to use a higher temperature of 43°C that would capture the full range of responses to heat stress within the trial period.

We used a water bath (Benchmark, MyBath 4L, model H2004) for these trials, to retain consistent heat and humidity (constant at 90% RH) across periodic (5 min) THS checks. Bees were taken from their holding container/colony and transferred to preheated (43°C ± 0.5°C) cylindrical glass vials (volume = 35.35 cm^3^) (Genesee Scientific, Flystuff, 32‐117BF) placed in a rack in a water bath (Benchmark, MyBath 4L, model H2004) (90% RH). Glass vials were weighed down with hex nuts (29.5 g) glued to the bottom of the vials and were cotton‐stoppered to allow for gas exchange and prevent CO_2_ buildup from respiration. Bees were checked every 5 min for symptoms of heat stupor by briefly lifting them from the water bath and flipping or shaking the vial as needed to assess if they could remain upright.

#### 2.2.2 Cross‐species analysis

We performed thermal tolerance assays on collected workers, males, and queens of wild and laboratory‐reared *B. impatiens* (*n* = 129 bees tested), *B. bimaculatus* (*n* = 45), *B. griseocollis* (*n* = 39), *B. perplexus* (*n* = 17), and the *B. vagans*/*B. sandersoni* (VS, *n* = 28) complex (Table A1). *B. vagans* and *B. sandersoni* are less common, are hard to tell apart morphologically, and occupy the same ecological niche in Pennsylvania higher elevation forests (Gratton et al., 2023), thus these were pooled to improve sample sizes. We identified *B. sandersoni* and *B. vagans* after running THS trials and found 31.6% of wild sampled bees were *B. sandersoni* (36.4% of workers, 37.5% of males) and all laboratory‐reared individuals were *B. sandersoni* (*n* = 4 colonies). If the analyses described below are run with these separately, the two species do not differ in THS (*B. vagans* [56.92 ± 16.78] and *B. sandersoni* [56.33 ± 29.72], *p* = .95) thus further justifying pooling them.

All statistical analyses were carried out in R version 4.2.1. We conducted an analysis of variance (ANOVA), both with and without interactions, to compare the effects of species, sex/caste (i.e., workers, males, and queens), and bee origin (laboratory‐reared or wild) on THS, followed by a Tukey's HSD test for multiple comparisons. For these two models (additive and interactive), we also calculated Akaike's information criterion, corrected for small sample size (AICc), using the AICcmodavg package (Mazerolle, 2017). While these models reveal the main effects, we also conducted separate analyses for visualization purposes on subsets of the data. In particular, we pooled wild and laboratory‐reared workers within each species and compared THS among species. We also compared laboratory‐reared *B. impatiens* workers, males, and queens, for which we had the most individuals of each sex/caste. We chose to exclude wild bees from this analysis due to potential confounding effects of origin on THS.

To assess species niche (i.e., latitude, climate, habitat type) relative to heat tolerance results, we used historical, georeferenced records of each *Bombus* species from GBIF (GBIF.org, 2023a, 2023b, 2023c, 2023d, 2023e, 2023f). For latitude, we filtered specimen records from the United States and Canada and only used specimen occurrence records east of 100.00° longitude to focus on eastern North America. From this, the 1% tails of the distribution were excluded to focus on the core range of each species. Within each of these ranges, we obtained average summer temperatures for the months when workers are primarily foraging—June, July, and August—aggregated over 30 years (1991–2020). Climate data was extracted from PRISM Climate Group (2020) using the prism package (Hart & Bell, 2015). We used separate ANOVA models followed by Tukey's HSD tests to assess both species differences in mean latitudinal distributions and summer mean temperatures. As PRISM data does not include climatic data for eastern Canada, Canadian specimens were not included in the temperature analysis. We also compare our results to data on altitudinal and habitat niche (forested hills, open valleys, and the transitional habitat between these) of each of these species in Pennsylvania (Gratton et al., 2023).

### 2.3 Time to heat stupor assays: *Bombus impatiens* assays of age, reproductive state, and body size

We assessed the effect of colony origin, age, reproductive state, body size, and humidity on thermal tolerance in *B. impatiens*. We focused on just *B. impatiens* because it had more robust sample sizes for these comparisons.

#### 2.3.1 Colony

We tested the effect of colony origin on THS in workers from five commercial colonies (*n* = 3–5 bees/colony) and three colonies reared from wild queens (*n* = 13–16 bees/colony; Table A1), using a two‐way ANOVA followed by a Tukey's HSD test, testing the effect of both colony and wild versus commercial origins. There was no significant variation in thermal tolerance with colony and thus we did not include colony identity in future models.

#### 2.3.2 Age

We compared THS among *B. impatiens* queens of different ages, drawn from three different commercial colonies: 4‐day‐old (*n* = 17), 7‐day‐old (*n* = 4), 10–12‐day‐old (*n* = 9), and 12–32‐day‐old (*n* = 17) (Table A2). For the first three age groups, callow queens were removed from their natal colonies daily, and individuals from the same colony were kept in a container together and provisioned with artificial nectar and pollen ad libitum until they reached the desired age. The 12–32‐day old cohort was removed from natal colonies at 2–20 days after adult eclosion and kept together prior to sampling. We also compared THS among two different age groups of *B. impatiens* workers. Seven‐day‐old (*n* = 12) and 14‐day old workers (*n* = 13) were sampled from three laboratory colonies reared from wild‐caught queens. Callow workers were removed from their natal colonies and placed in mini‐colonies (a few individuals together per container) provisioned with artificial nectar and pollen ad libitum until they reached the desired age. The effect of age on mean THS for both queens and workers were tested separately using ANOVAs followed by Tukey's HSD tests.

#### 2.3.3 Ovarian activation in queens

We sought to test whether ovarian and physiological state may impact heat tolerance in *B. impatiens* queens. Carbon dioxide (CO_2_) narcosis in bumble bees is known to induce a post‐diapause reproductive state in bumble bees and trigger them to initiate egg production (Amsalem & Grozinger, 2017). We thus tested bees with and without CO_2_ narcosis to test effects of diapause, and bees at 2 days post‐narcosis, which is prior to or during early ovarian activation/egg development for CO_2_‐treated bees, and 7 days post‐narcosis, when ovarian activation/egg development is likely already completed for CO_2_‐treated bees (Amsalem & Grozinger, 2017), to examine effects of ovarian activation state. We administered CO_2_ to 17 *B. impatiens* queens reared from two commercial colonies that were between 12‐ and 21‐days‐old age by placing bees in plastic boxes that were sealed except for a small hole in the top where we administered CO_2_ through a hose at low pressure for 1 min, which is enough time for bees to enter CO_2_ narcosis. The hole was immediately taped over, sealing in the gas where the bees remained for 30 min. They were then placed into individual ventilated containers, where they emerged from their narcosis, and were provisioned with pollen patties and artificial nectar ad libitum. Another 17 *B. impatiens* queens of the same age group that did not undergo CO_2_ narcosis were used as controls. We divided treated and untreated bees into two subgroups in a fully crossed design: 2‐day post‐CO_2_ narcosis/2‐day control (*n* = 8 each) and 7‐day post‐CO_2_ narcosis/7‐day control (*n* = 9 each) (Table A2). After queens went through THS trials, queens were dissected, and ovary activation was measured by averaging the length of the largest terminal oocytes from each ovary, plus the largest remaining oocyte from one side (cf. Amsalem & Grozinger, 2017). We used ANOVAs followed by Tukey HSD tests to assess whether there are differences in mean THS between CO_2_ narcosis treatments and ovary activation (average terminal oocyte length). To further test the effects of ovarian activation on heat stress response, we performed a linear regression model comparing THS against average terminal oocyte length across all samples. Finally, some queens had started laying eggs, so we performed ANOVAs to test the effects of presence/absence of brood on mean THS and examined whether bees with brood indeed had more ovary activation.

#### 2.3.4 Body size

To test the effect of body size on heat tolerance, intertegular distances (ITD) were measured in 71 *B. impatiens* workers that underwent THS trials. ITD is a commonly used metric to assess body size in bumble bees and other bees and is considered a reliable metric of body size (Cane, 1987; Hagen & Dupont, 2013). ITD was measured with a dissection microscope ocular reticle and converted to millimeters for analysis. We did separate analyses for workers reared from wild‐caught queens (*n* = 55) and for wild workers (*n* = 17). For each group of workers (laboratory‐reared and wild), we used a simple linear regression model to test the effect of ITD on THS. The data for laboratory‐reared workers appeared somewhat curvilinear, so we also ran a second‐order polynomial regression on this data. We then compared this model to our linear regression for laboratory‐reared workers using AICc. We retained the linear regression model since it had the lower AICc (172.7 compared to 176.0).

### 2.4 Humidity and temperature interactions in THS

To test the interaction between temperature and humidity, we first conducted an experiment on commercial *B. impatiens* workers (*n* = 4 colonies; three replicates per colony/condition; *n* = 12 bees/condition) at three different humidity conditions: 20%, 50%, and 70% RH (range ± 4% during trials). These humidities were chosen to span dry, average, and higher humidity conditions. Bees were taken from their natal colony, placed into separate dry incubators (Thermo Scientific, 371 L) set at 43°C (±2°C during trials), and checked every 5 min for heat stupor.

As the temperature fluctuated quite a bit during the first experiment, likely due to our regular opening of the door, we ran a second experiment using a single device for all specimens, seeking to control environmental conditions more. Given the results from the first experiment, we focused only on the highest and lowest humidities (20% and 70%). For this, we drew 10 workers randomly for each condition (20% [17%–29%] and 70% [67%–77%]) from a single commercial parent colony that was generating only workers, and we also drew 10 queens (aged 10‐ to 21‐days‐old) from a different commercial colony that was in a queen‐producing stage. Bees were moved into a small (23.5 cm × 27.5 cm × 36 cm) incubator (Vevor, model: XHC‐25) that was set to 43°C (actual temperature: 42.9–43.5°C at 20% and 42.4–42.7°C at 70%), set within a walk‐in incubator at similar humidity to the treatment and at 28°C, and assessed for THS. We compare these data with THS results from *B. impatiens* workers (*n* = 69) run at a stable 43°C and 90% RH in a water bath. Note that only for humidity trials did we use incubators; water baths were used for all other trials.

For the humidity trial analysis, given that much of our data were truncated to 3 h due to the great number of heat resistant bees at low humidity, we ran a GLM binomial regression to assess the proportion of bees that entered heat stupor as a function of humidity (20% vs. 70% RH) and caste (workers and queens). We also ran an ANOVA followed by Tukey tests on both humidity datasets separately, testing the effects of humidity treatment for the first experiment and both humidity and caste for the second.

## 3 RESULTS

### 3.1 Cross‐species analysis (species, caste, and origin) of heat tolerance

#### 3.1.1 Species

We found a strong effect of species (*p* < .001) on mean THS in our full model (Table 2). Overall means in THS by species placed *B. griseocollis* (96.9 min ± 44.4 [SD]) as the most heat tolerant, followed by *B. impatiens* (80.8 ± 30.1), *B. perplexus* (71.8 ± 40.0), and *B. bimaculatus* (71.1 ± 35.2), which were not statistically different from one another, and *B. vagans*/*sandersoni* (56.6 ± 24.2) as the least heat tolerant. Pairwise comparisons among species (Tukey's HSD Test) (Table 3) revealed *B. griseocollis* to have a higher THS than *B. impatiens*, *B. perplexus*, *B. bimaculatus*, and *B. vagans*/*sandersoni*, while *B. impatiens* have a higher mean THS than *B. vagans*/*sandersoni*. The interactive model had a slightly lower AICc (2477) compared to the additive model (2481), however, we report statistics from both models in Table 2. A subset model run only on workers likewise found the same patterns of significance, except that *B. perplexus* was not significantly different from *B. griseocollis* (*p* = .161): *B. griseocollis* (117.9 min ± 37.2 [SD] > *B. impatiens* [87.5 ± 29.4] = *B. perplexus* [78.8 ± 53.4] = *B. bimaculatus* [77.7 ± 35.2] > *B. vagans*/*sandersoni* [55.9 ± 21.2]) (Figure 1B, Table 3). This THS order tracks the mean latitude, climatic distribution, and habitat preferences for these different species based on species distributions obtained from GBIF (Figure 1B). Species varied significantly in median latitude (*F*_4,86,440_ = 2844, *p* < .001) with *B. impatiens* (40.78) < *B. bimaculatus* (41.0) < *B. griseocollis* (41.17) < *B. perplexus* (41.81) < *B. vagans*/*sandersoni* (43.65), while the southern and northern latitudinal limits, respectively, show *B. impatiens* (28.28, 44.93) < *B. griseocollis* (30.84, 46.98) </> *B. bimaculatus* (33.25, 46.87) </> *B. perplexus* (35.57, 49.69) < *B. vagans*/*sandersoni* (35.75, 48.78). Similarly, when these georeferenced datapoints are used to extract 30‐year normal June–August temperatures, we see a highly significant difference in mean temperatures by species (*F*_4,83,684_ = 4114, *p* < .001) and between each species pair (Tukey *p* < .001 for all comparisons) where *B. griseocollis* (22.32°C) > *B. impatiens* (22.21°C) > *B. bimaculatus* (21.54°C) > *B. perplexus* (20.95°C) > *B. vagans*/*sandersoni* (19.76°C) (Figure 1B). This matches well with the recognized differences in distribution in Pennsylvania, where *B. impatiens* and *B. griseocollis* are more abundant in open valleys, *B. vagans*/*sandersoni* are more abundant in hilled forests and *B. bimaculatus* and *B. perplexus* are most abundant at the interface of these habitat zones.

**TABLE 2:** ANOVA results for significance in time to heat stupor (THS) across conditions, including statistics for the additive MLRM on THS across species, sex/caste, and origin (top) and for the interactive multivariate model (bottom).

Variables	df	*F* value	Pr(>*F*)
Species	4	8.38	<.001***
Origin	1	21.18	<.001***
Sex/caste	2	21.6	<.001***
Residuals	248		
Species	4	9.24	<.001***
Origin	1	23.36	<.001***
Sex/caste	2	23.82	<.001***
Species*origin	4	4.56	.001**
Species*sex/caste	8	1.88	.066
Origin*sex/caste	2	0.924	.398
Species*origin*sex/caste	3	2.476	.062
Residuals	231		

*Note*: Bold type indicates statistical significance.

**p‐value between .01 and .001. ***p‐value less than .001.

**TABLE 3:** Results from the post hoc Tukey analyses by species (all caste/sex and workers only), sex/caste (for all species and *Bombus impatiens* only), and queens of different ages.

Comparison	THS diff. [95% CI]	*p*‐Adjusted	THS diff. [95% CI]	*p*‐Adjusted
All caste/sex			Workers only	
gris‐bimac	25.79 [7.56, 44.01]	**.001**	40.18 [14.98, 65.37]	**<.001**
imp‐gris	−16.10 [−31.26, −0.94]	**.031**	−30.33 [−51.55, −9.11]	**.001**
perp‐gris	−25.16 [−49.24, −1.07]	**.036**	−39.11 [−86.72, 8.51]	.161
vs‐gris	−40.32 [−60.85, −19.79]	**<.0001**	−61.92 [−90.88, −32.96]	**<.001**
imp‐bimac	9.69 [−4.80, 24.17]	.355	9.85 [−9.11, 28.81]	.607
perp‐imp	−9.06 [−30.45, 12.34]	.772	−8.78 [−53.41, 35.85]	.983
vs‐imp	−24.21 [−41.50, −6.92]	**.001**	−31.59 [−55.33, ‐7.85]	**.003**
bimac‐perp	0.63 [−23.04, 24.30]	1.0	1.07 [−45.58, 47.72]	1.0
vs‐bimac	−14.53 [−34.56, 5.51]	.273	−21.74 [−49.09, 5.61]	.190
vs‐perp	−15.16 [−40.64, 10.33]	.477	−22.81 [−71.60, 25.98]	.697
Caste‐all spp.			Caste‐*B. impatiens*	
Queen‐male	−30.90 [−46.27, −15.52]	**<.001**	−33.44 [−55.29, −11.60]	**<.001**
Worker‐male	10.50 [0.22, 20.78]	**.044**	6.14 [−12.61, 24.90]	.716
Worker‐queen	41.40 [27.41, 55.38]	**<.001**	39.59 [24.91, 54.27]	**.001**
Queen age				
7–4 day	37.57 [8.62, 66.53]	**.006**		
10–4 day	54.38 [32.90, 75.86]	**<.001**		
12+−4 day	22.64 [4.78, 40.52]	**.008**		
10–7 day	16.80 [−14.50, 48.11]	.485		
7–12+ day	14.93 [−43.88, 14.03]	.520		
10–12+ day	31.73 [−53.21, −10.25]	**.002**		

*Note*: 10 day is 10–12 day.

Bold type indicates statistical significance.

Abbreviations: bimac, *Bombus bimaculatus*; gris, *Bombus griseocollis*; imp, *Bombus impatiens*; min, minutes; perp, *Bombus perplexus*; vs, *Bombus sandersoni* + *Bombus vagans*.

#### 3.1.2 Caste differences

THS was different by sex/caste (*p* < .001) (Table 2) with the greatest difference in queens from workers and males (Table 3, *p* < .001) and a weakly significant difference between workers and males (*p* = .044). Workers had the highest THS across species (mean = 86.37 min ± 34.91 [SD]) followed closely by males (75.50 ± 29.57), and then by queens (44.35 ± 25.68) (Figure 1A). There was a marginally significant interaction between sex/caste and species (*p* = .066). *B. griseocollis* had a particularly marked difference between workers (mean THS = 117.86 min ± 37.17) and males (72.33 ± 36.59) (*p* < .001).

When looking at sex/caste difference just within *B. impatiens*, we found a significant difference in mean THS (*F*_2,93_ = 20.67, *p* < .001), with higher THS in workers (mean 82.54 min ± 25.31 [SD]) than queens (43.06 ± 16.46) (*p* < .001) and males (76.5 ± 17.49) than queens (*p* = .012), but not between workers and males (*p* = .716) (Figure 1C; Table 3).

#### 3.1.3 Origin of bees

We found a significant difference between laboratory‐reared and wild bees overall (*p* < .001), an interactive effect of species and origin (wild‐ vs. laboratory‐reared) (*p* = .002) (Table 2), and a trend for interactions of origin with sex/caste and species (*p* = .062). Across species, wild‐caught workers had higher THS on average than laboratory‐reared workers with the exception of *B. griseocollis*, where the opposite pattern was observed (Figure 1A).

**FIGURE A2:**
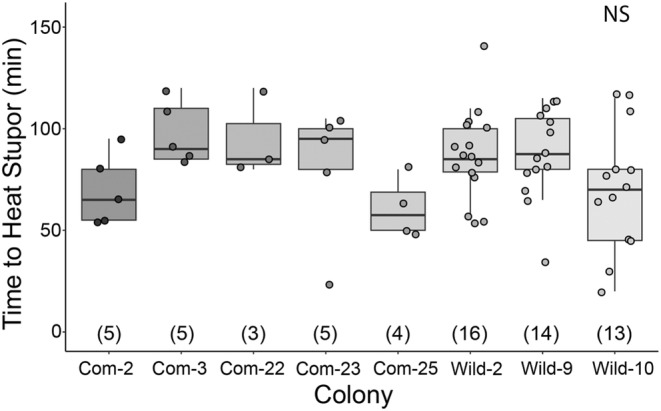
Comparisons of time to heat stupor in *Bombus impatiens* workers between colonies, including commercial colonies (5) and colonies reared from wild‐caught queens (3). There was no significant colony effect, although there were some differences in means that may have been significant with more sample size per colony. Differences were unrelated to whether bees came from commercial or wild‐laboratory‐reared colonies, thus, these bees are treated the same in analyses.

With our analyses in *B. impatiens* workers, there was no significant colony effect (*n* = 8 colonies) on mean THS (*F*_6,56_ = 1.85, *p* = .105) (Figure A2). There was no difference between colonies obtained commercially (mean = 80.68 min ± 24.17 [SD]) from those reared from local queens (82.73 min ± 25.90) (*F*_1,56_ = 0.10, *p* = .749).

### 3.2 Effect of age, physiology, and body size in *Bombus impatiens*

#### 3.2.1 Age trials

**FIGURE 2:**
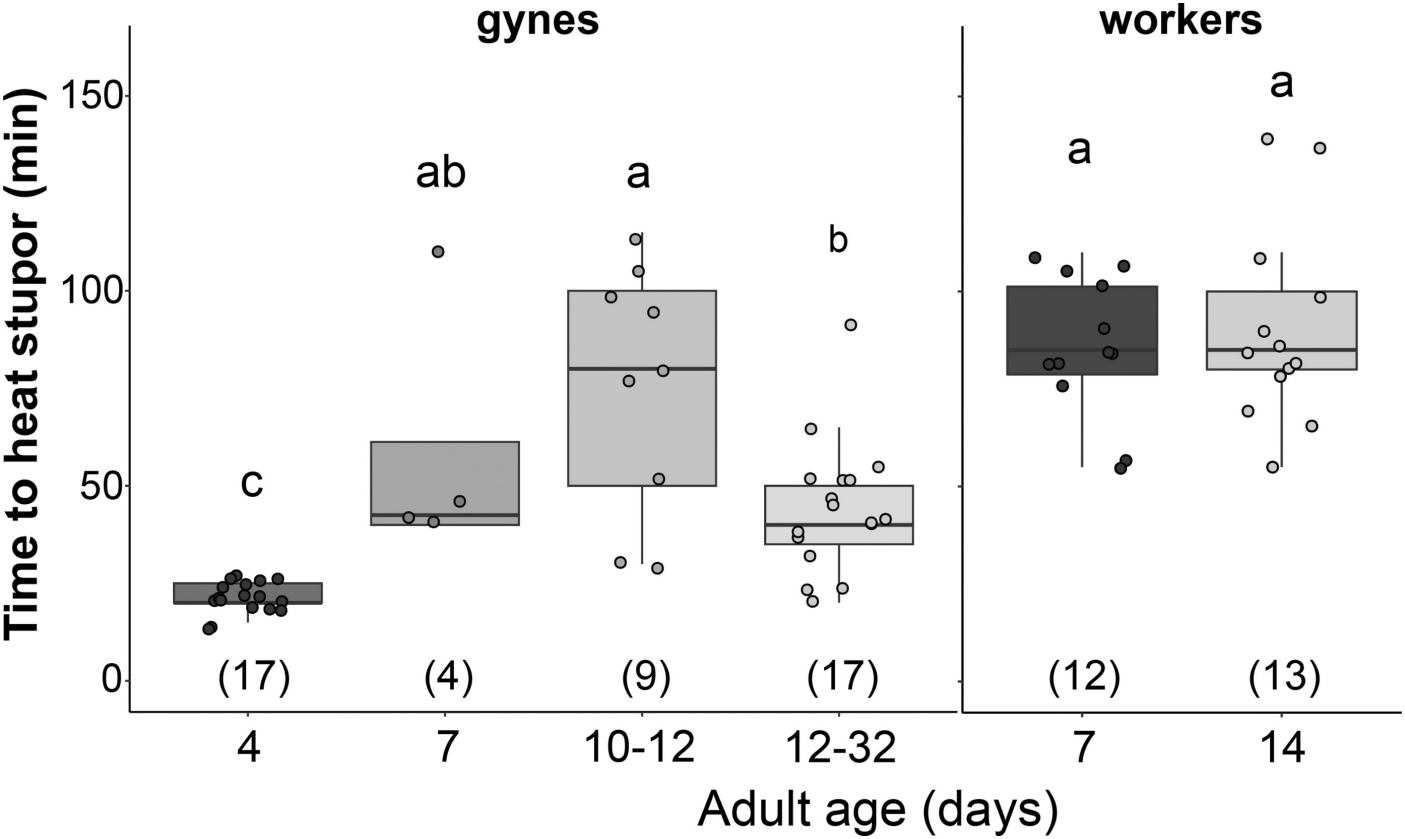
Differences in mean time to heat stupor relative to age in *Bombus impatiens* queens and workers. Individuals are age‐staged from commercially reared *B. impatiens* colonies. Significance letters represent differences within each caste (queens or workers). Numbers in parentheses are sample sizes. min, minutes.

The ANOVA showed a significant effect of age on mean THS (*F*_3,43_ = 16.35, *p* < .001) in queens (Figure 2). Queens aged 12–32 days (mean = 43.82 min ± 16.63), 10–12 days (75.56 ± 32.05), and 7 days (58.75 ± 34.25) had higher THS than 4‐day‐old queens (21.18 ± 3.32), and the 10–12‐day‐old queens had higher mean THS than 12–32‐day‐old queens (Table 3). The 7‐ and 12–32‐day‐old queens showed similar THS to spring queens sampled from the wild (Figures 1 and 2), while 4‐day‐old queens had lower THS, and 10–12‐day‐old queens had higher THS than wild‐caught spring queens. We found no significant difference in mean THS between 7‐day‐old (85.42 ± 18.15) and 14‐day‐old (90.38 ± 25.20) workers (Figure 2).

#### 3.2.2 Ovarian activation in queens

**FIGURE 3:**
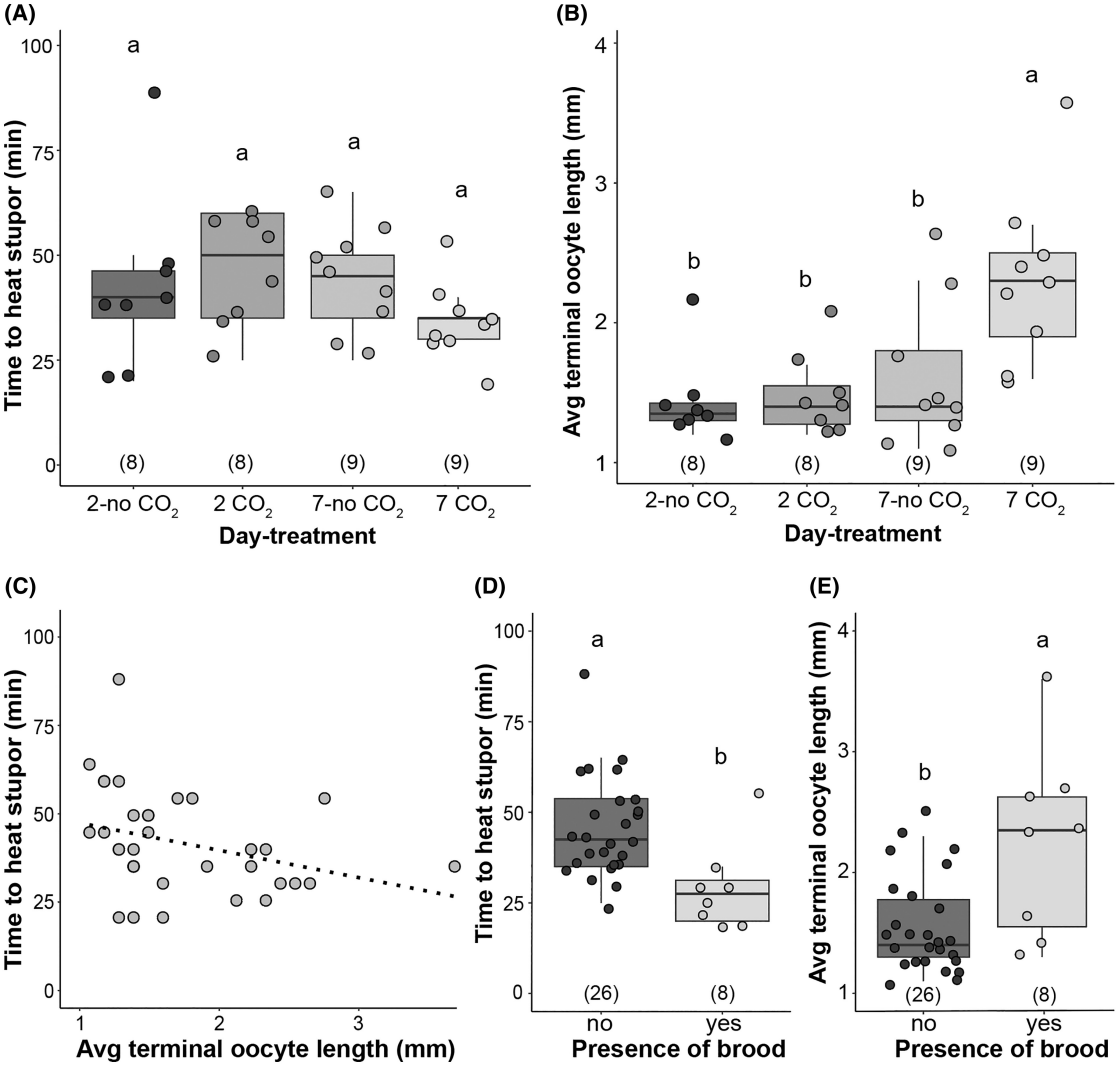
Effect of queen physiology (diapause condition and ovary activation) on time to heat stupor (THS). (A) Effect of CO_2_ narcosis treatment and age on mean THS in commercially reared *Bombus impatiens* queens. CO_2_ narcosis‐treated bees represent a physiological state akin to having gone through diapause (spring queens) while those without it represent the state of pre‐diapause fall queens. 2‐day treatments represent an age typically too early for ovaries to have developed and 7 days should be sufficient to allow ovary activation for diapaused queens. (B) Average terminal oocyte length (a measure of degree of ovary activation) by CO_2_ treatment, showing that 7‐day, CO_2_‐treated queens had greater ovarian activation. (C) THS regressed against ovary activation across all CO_2_‐treated bees. CO_2_ was administered to queens aged 12–21 days (adjusted *R* ^2^ = .077, *p* = .062). (D) THS regressed against presence/absence of brood, showing that queens with brood have lower THS than queens without brood. (E) Average terminal oocyte length regressed against presence/absence of brood, showing that presence of brood is positively associated with ovary activation. Sample sizes for each condition are indicated in parentheses.

CO_2_ narcosis did not result in a significant effect on mean THS (*F*_3,30_ = 1.11, *p* = .36; Figure 3A). However, CO_2_ narcosis was effective at initiating ovary activation; average terminal oocyte length was significantly correlated with narcosis treatment (*F*_3,30_ = 6.02, *p* = .002) (Figure 3B), with 7‐day‐post‐CO_2_ narcosis bees having higher ovary activation than all other treatment groups, which were not significantly different from each other. Overall, degree of ovary activation showed a trend toward a correlation with THS (*F*_1,32_ = 3.74, *p* = .062) (Figure 3C), whereby greater ovary activation was associated with decreased THS. Queens that were egg‐laying had higher rates of ovary activation (*F*_1,32_ = 10.7, *p* = .026), and showed lower mean THS (*F*_1,32_ = 9.06, *p* = .005) (Figure 3D,E).

#### 3.2.3 Body size

**FIGURE 4:**
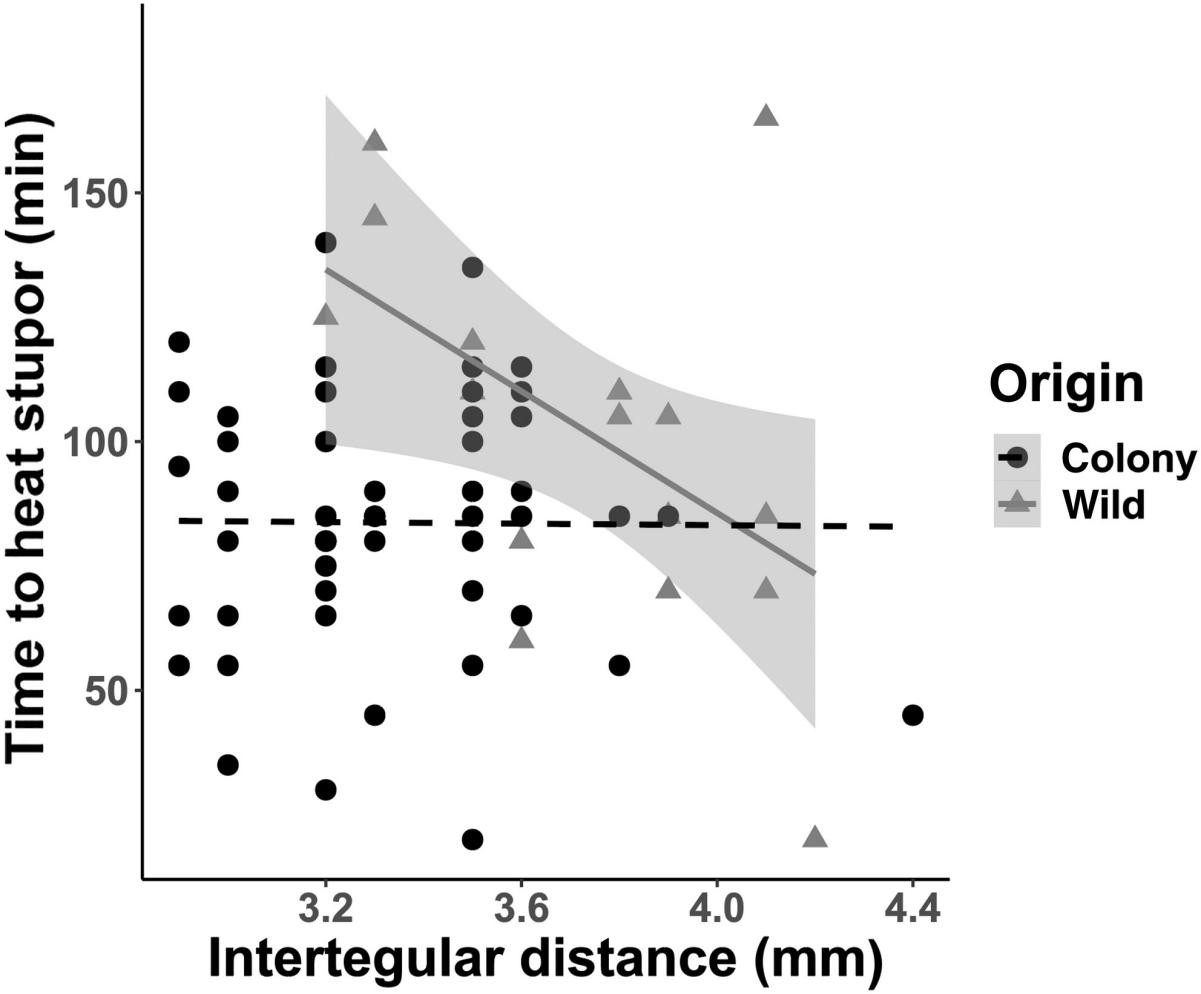
Time to heat stupor regressed against body size (intertegular distance in millimeters) for wild (triangles) and laboratory‐reared (circles) *Bombus impatiens* workers. THS was correlated with body size in wild workers (adjusted *R* ^2^ = .26, *p* = .0363) but not in laboratory‐reared workers (adjusted *R* ^2^ = −.0188, *p* = .95).

We found larger body size was associated with decreased THS for wild *B. impatiens* workers (*R*‐squared = .26, *F*_1,15_ = 5.29, *p* = .036), but there was no effect of body size on laboratory‐reared workers (Figure 4). On average, wild workers had a larger body size than laboratory‐reared workers (*p* = <.001).

### 3.3 Evaluating the interaction of humidity and temperature on THS in *Bombus impatiens*

**FIGURE 5:**
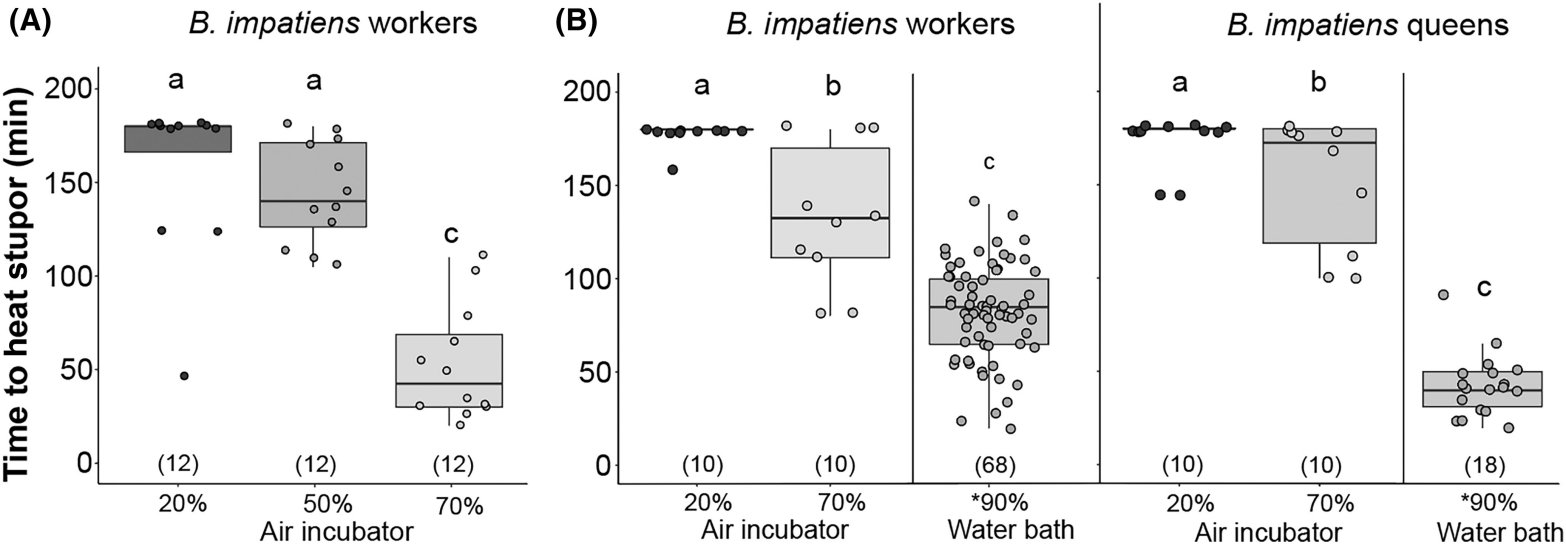
Time to heat stupor (THS) relative to humidity for commercially reared *Bombus impatiens* workers and queens. (A) Experiment 1 with workers. (B) Experiment 2 with both workers and queens. Significance from Tukey tests is indicated. Sample sizes for each condition are indicated in parentheses. *The 90% condition involved water baths while the remainder of the treatments were performed in air incubators. Differences in results by experiment are likely due to the way temperature was calibrated for each experiment which resulted in slightly higher temperatures for Experiment 1.

In our initial humidity experiment evaluating the effect of humidity (20%, 50%, and 70% RH) on the THS of worker *B. impatiens* bees, we found significant differences in mean THS between 20% and 70% RH (*p* < .001, 95% CI = [−140.77, −72.56]), 50% and 70% RH (*p* < .001, 95% CI = [−126.19, −57.98]), but not between 20% and 50% RH (Figure 5A). Overall, heat tolerance declined with increasing humidity, with more dramatic declines around 70%. Many bees in the 20% and 50% RH trials were heat resistant, which may explain in part the nonsignificant results between these two groups.

In the second experiment, bees at 20% RH were more heat resistant than bees exposed to 70% RH (*p* < .001, 95% CI = [−0.81, −0.29]), with no significant effect of caste (queen vs. workers). Humidity had an effect on heat resistance in both workers (*p* = .004, 95% CI = [−0.98, −0.22]) and queens (*p* = .018, 95% CI = [−0.90, −0.10]). All three humidity levels (20%, 70%, and 90%) support a highly significant effect of humidity for both workers (*F*_2,85_ = 69.39, *p* < .001) and queens (*F*_2,35_ = 147, *p* < .001) (Figure 5B). Treatments 20% and 90%, and 70% and 90% for both queens and workers differed in THS (*p* < .001) and THS at 20% and 70% were also different for queens (*p* = .045) and workers (*p* < .001), with THS being greater at lower humidity.

## 4 DISCUSSION

Prolonged hyperthermic stress in bumble bees can have lethal as well as sublethal effects on bee health, such as limiting forage time and flight ability (Hemberger et al., 2023), and impacting colony growth and reproduction (Vanderplanck et al., 2019). The frequency and duration of heatwaves across the globe are predicted to increase due to climate change (Domeisen et al., 2023), and thus many bumble bee populations are more likely to experience hyperthermic stress in the future. To better understand how these bees will respond to this change, we have provided data toward understanding how different species respond to heat stress and which physiological, morphological, and life‐history factors make them more vulnerable. We found that species, caste and sex, origin, age, ovary activation, body size, and relative humidity all play a role in how the bumble bee species in our study region respond to heat stress, but to different extents. Most notably, we found that bumble bee species vary in heat tolerance in a way that matches their habitat distributions, suggesting that heat sensitivity likely impacts their range. We find that queens are most vulnerable, but that their vulnerability is dependent on age and reproductive status. Our field to laboratory comparisons suggest that some of the exceptional variability we find in heat tolerance in the field may be due to physiological status or environmental conditions. Our data further highlight the strong effect humidity plays in thermal resilience and the need to consider humidity and temperature interactions in future work. Our results identify points of vulnerability in these bees to consider when addressing decisions and policy regarding bumble bee conservation, especially in the context of climate change.

### 4.1 Thermal tolerance of species tracks their distribution

Martinet et al. (2015) and Martinet, Dellicour, et al. (2021), in comparing bumble bees across the globe, found differences in heat tolerance that reflected their respective ecoregions. Other work in montane regions have found that bumble bee altitudinal ranges are correlated more with cold tolerance than with heat tolerance (Pimsler et al., 2020). Large‐scale climate modeling lends support to heat vulnerability driving shifting ranges, range contraction, and species loss, suggesting natural variation in heat tolerance may be a reason that some species are particularly vulnerable (Kerr et al., 2015; Sirois‐Delisle & Kerr, 2018; Soroye et al., 2020). We find a clear trend in inferred heat tolerance that matches the temperature of zones these species occupy. Species with higher mean THS have more southerly median latitudinal distributions, warmer climatic distributions, and prefer valleys to forested and higher elevation habitats. Specifically, we found that *B. griseocollis* and *B. impatiens* were more heat tolerant and occur in the warmer latitudes/climates and in Pennsylvania valleys, while the higher latitude forest‐restricted species *B. vagans*/*sandersoni* were the least heat tolerant, and *B. bimaculatus* and *B. perplexus*, which have been found in edge habitat between these zones and were intermediate in their climatic and distributional means had more intermediate heat tolerance. Bumble bees may also vary in nesting position relative to the ground surface, which can impact the ability to escape heat and the overall exposure to ambient temperatures. For the species studied here, *B. impatiens* is a primarily underground nester, *B. bimaculatus*, *B. vagans*, and *B. perplexus* are thought to be mostly underground nesters with some surface nesting, and *B. griseocollis* is primarily a surface nester (Husband, 1977; Plath, 1922, 1927, 1934); thus, *B. griseocollis* would have nests that are less buffered from heat waves. *B. griseocollis* did show slightly higher heat tolerance than *B. impatiens*, which was not well matched by differences in the thermal occupancy between them.

These results suggest that some species in a community are more vulnerable to heat stress than others, and that the increased frequency and duration of heat events under climate change may disproportionately impact bumble bee species with cooler climatic distributions that typically inhabit forests and more northerly latitudes. For example, the relatively high heat tolerance of *B. impatiens* and *B. griseocollis* may be one reason these species' populations are increasing proportionally in the Eastern United States and Midwest in the last several decades (Averill et al., 2021; Jacobson et al., 2018; Novotny et al., 2021) compared to the species that occupy cooler areas. *B. vagans* and *B. sandersoni* may be especially impacted since they not only have cooler climatic distributions, but have suffered recent population declines in parts of their ranges (Colla et al., 2012; Grixti et al., 2009; Jacobson et al., 2018). These results can help inform conservation management decisions by prioritizing species that are particularly sensitive to extreme heat events and are already of conservation concern.

### 4.2 The environment influences variance in thermal tolerance

We found that wild bees have higher tolerance to hyperthermic stress than bees reared in the laboratory (with the exception of *B. griseocollis*), indicating that environmental conditions may be involved in heat stress resilience. In contrast, Gonzalez et al. (2022) found laboratory‐reared bumble bees to have slightly higher thermal tolerance. Although it would require further testing, laboratory‐reared bees were fed honey bee‐collected pollen which did not allow the bees to seek out their nutritional optima. For example, honey bee pollen is lower in protein:lipid ratios compared to what bumble bees obtain when foraging in the wild (Vaudo et al., 2018). This could have reduced food quality and have negative effects on heat resilience at the colony (Vanderplanck et al., 2019) and individual (Quinlan et al., 2023) level. Other factors besides nutrition may also vary between field and laboratory, such as pathogen levels, optimal hydration, and ability to attain optimal nest conditions: these factors need further study to fully understand their impact on thermal tolerance. Furthermore, laboratory‐reared bees may have included nurse bees, while wild‐collected bees are only foragers. While wild bees will have experienced greater temperature fluctuations during their lifetimes than laboratory‐reared bees, recent studies demonstrate that prior experience of thermal stress does not result in increased thermal tolerance through priming (Quinlan et al., 2023; Sepúlveda & Goulson, 2023).

### 4.3 Sex and caste impact thermal tolerance

Across all species, and when analyzing *B. impatiens* alone, queens had lower THS than workers and males. The relatively high sensitivity of queens to hyperthermic stress in the laboratory appears to make them particularly vulnerable to extreme heat compared to workers and males. This effect, however, was only observed in our water bath trials where relative humidity was 90%. At 70% RH, queens were not different than workers, indicating an interaction between caste and humidity.

In our assays, males were slightly less heat tolerant than workers, although the significance was marginal, and this showed a trend toward being dependent on species. Prior work found no difference in CT_max_ between males and workers in three species in the Rocky Mountains (Oyen et al., 2016) and no differences in CT_max_ between worker and males among four *Bombus* species native to Columbia (Gonzalez et al., 2022). There is temporal overlap between male and queen emergence, both leaving the nest in the heat of late summer, putting them at similar risk of encountering the same extreme heat events. While males were less sensitive to heat stress (had higher THS) than queens, sperm viability has been documented to decline in male bumble bees exposed to elevated temperatures that fall far below the temperatures tested here (Campion et al., 2023; Martinet, Zambra, et al., 2021), thus cross‐generational effects must also be considered in their heat adaptation and vulnerability.

### 4.4 Age effects on thermal tolerance depend on caste

Our *B. impatiens* trials show that queens differ in their thermal tolerance by age. Four‐day‐old queens are the most vulnerable to heat stress, followed by a steady increase in THS as they age until around 12 days where their levels reached the higher levels typically observed for workers, after which they decline to the levels we typically observed in wild queens/queens. This suggests that queens change physiologically as they age, which impacts their response to heat stress. Oyen and Dillon (2018) similarly found that 4‐day‐old workers were less heat tolerant than 3‐ or 7‐day‐old workers, suggesting this may be a vulnerable stage. The peak of heat tolerance in queens at 12 days is intriguing in its biological correlates: Queens are thought to leave the natal nest to mate and find a hibernaculum at 7–12 days‐old (Tasei et al., 1998; Treanore et al., 2021) and then likely enter cooler substrates for diapause thereafter. Thus, thermal tolerance may align with the period when queens are most exposed to thermal stress.

We have also expanded on data by Oyen and Dillon (2018) by examining the effects of age in older worker bees. We found no effect of age between 7‐ and 14‐day olds on heat tolerance, despite 14‐day olds being near the point of mortality (only one 21‐day‐old worker survived and was tested, but it had a similar thermal tolerance to 7‐ and 14‐day olds).

### 4.5 Ovary activation state impacts thermal tolerance

One factor that may make queens more vulnerable to heat is their investment in reproduction. Queens increase ovary activation 1–2 weeks after diapause or CO_2_ narcosis (Amsalem, Galbraith, et al., 2015). Ovary activation in queens is associated with physiological changes such as increases in juvenile hormone (JH), ecdysteroids, and body fat metabolism (Amsalem, Galbraith, et al., 2015; Bloch et al., 2000). These hormones have been shown to respond to temperature (González‐Tokman et al., 2020) and regulate thermal tolerance stress responses in insects (e.g., in *D. melanogaster*; Gruntenko & Rauschenbach, 2008). Our data did not support that shifting to a physiological state akin to diapause influences thermal tolerance, as THS was not significantly different between 2‐day and 7‐day post‐CO_2_ narcosis‐treated queens and controls. It did, however, support that shifts in ovarian activation and brood production post‐narcosis impact thermal tolerance.

### 4.6 Body size effects are context‐dependent

Body size in principle, given the surface to mass ratio and ability to dissipate heat, should relate to thermal tolerance, with smaller bees better able to handle the heat. Prior research in bumble bees have found mixed effects of body size on thermal tolerance, from no effect of body size to effects in some species (Table 1). We found larger body size was associated with lower THS for wild *B. impatiens* workers but no effect of body size on laboratory‐reared workers. However, since we randomly selected workers from laboratory colonies for our trials, we likely picked some bees that were nurse bees that are restricted to in‐nest activities and some that are “foragers,” while only foragers were sampled in the wild. Also, in line with nurse bees being generally smaller than foragers (e.g., Holland et al., 2021), wild workers were larger on average than laboratory‐reared workers and the smallest individuals were from laboratory‐reared individuals. Our findings could reflect interactions of size with ovarian activation/physiology of workers with different tasks (e.g., foragers and nurses) and nutritional state. Foragers may also be healthier bees, thus allowing better assessment of effects of body size without confounding factors (nutrition and division of labor). These correlations suggest that declines in body size of collected bumble bees over time (Gérard et al., 2022; Nooten & Rehan, 2020) should consider potential roles of climate in this shift.

### 4.7 Humidity impacts thermal tolerance

Prior studies on bumble bee thermal tolerance have focused on temperature without mentioning potentially confounding effects of humidity (Gonzalez et al., 2022; Hamblin et al., 2017; Maebe et al., 2021; Martinet, Dellicour, et al., 2021; Oyen et al., 2016; Oyen & Dillon, 2018; Pimsler et al., 2020). We found that humidity can have strong effects on thermal tolerance and thus that humidity needs to be carefully controlled in these thermal experiments. Heat tolerance declined with increasing humidity (20%–70%–90% RH) for both workers and queens, with the effects being most notable at the highest humidity levels. A likely possibility for this association is that as air becomes more saturated at higher humidity levels, bees are less able to use evaporative cooling to bring down their body temperature. While convective cooling is thought to be the primary means by which bumble bees cool themselves, such as through shunting of hemolymph to dissipate heat through the abdomen (Heinrich, 2004), evaporative cooling is another means by which some insects survive otherwise lethal temperatures (Prange, 1996). Evaporation occurs through tracheal ventilation and in bees cooling through mouth regurgitation of nectar is purported (Heinrich, 2004; Prange, 1996). Evaporative cooling has been observed to be more efficient in dry air than in saturated air in a number of insects, including bumble bees (Church, 1960; Prange, 1996). Bumble bee wet body mass has also been found to be more related to heat tolerance than dry mass, suggesting hydration may be important to thermal tolerance in these bees (Martinet, Dellicour, et al., 2021). The interaction between humidity and caste, whereby the reduced thermal tolerance of queens was supported only at higher humidity, may suggest that queens rely on evaporative cooling more than workers. Given these strong effects, there is a need for research on the physiological effects of humidity on thermal tolerance.

## 5 CONCLUSION

Our data support heat tolerance as a factor impacting where species can occur, both in terms of geographic ranges and habitat preferences, suggesting these bees are limited in part by heat sensitivity and this may play a role in their declines. We also show that queens are more vulnerable to heat stress than workers and males and especially when they are newly emerged, have active ovaries, and are producing brood. This supports a role of physiological and developmental state in not only influencing thermal response but also points to a bottleneck of climate vulnerability in the founding queen. The observed effect of body size in wild foragers suggests that selection on body size should be considered in the context of climate warming. Finally, we show that humidity has a strong effect on thermal tolerance suggesting that humidity and temperature need to be considered together in assessing climate impacts. Future research on thermal impacts in these bees should assess if adaptation is driving distant populations of the same species toward variable responses to heat stress, mechanisms behind how humidity and differences in physiology influence thermal tolerance, and the role of landscape health, such as pathogens and nutrition, in thermal resilience. Our work will be foundational to these future studies and will contribute to devising conservation strategies for bumble bees that continue to experience more extreme temperatures.

## AUTHOR CONTRIBUTIONS

**Cody Feuerborn:** Conceptualization (supporting); data curation (lead); formal analysis (lead); investigation (lead); methodology (equal); software (lead); visualization (lead); writing – original draft (lead); writing – review and editing (supporting). **Gabriela Quinlan:** Formal analysis (supporting); software (supporting); writing – review and editing (supporting). **Rachael Shippee:** Data curation (supporting); investigation (supporting); methodology (supporting); writing – review and editing (supporting). **Tori L. Strausser:** Data curation (supporting); investigation (supporting); methodology (supporting); writing – review and editing (supporting). **Tatiana Terranova:** Investigation (supporting); writing – review and editing (supporting). **Christina M. Grozinger:** Conceptualization (supporting); funding acquisition (supporting); methodology (supporting); project administration (supporting); writing – review and editing (supporting). **Heather M. Hines:** Conceptualization (lead); funding acquisition (lead); methodology (equal); project administration (lead); visualization (supporting); writing – original draft (supporting); writing – review and editing (lead).

## FUNDING INFORMATION

This work was funded by the Agricultural Resource Center funding from the Pennsylvania Department of Agriculture to the Center for Pollinator Research at Pennsylvania State University (154185). GMQ was supported by the NSF Postdoctoral Research Fellowship in Biology Program under Grant No. 2109109. Any opinions, findings, and conclusions or recommendations expressed in this material are those of the authors and do not necessarily reflect the views of the National Science Foundation. This work was supported by the USDA National Institute of Food and Agriculture and Hatch Appropriations under Project #PEN04716 and Accession #1020527.

## CONFLICT OF INTEREST STATEMENT

The authors declare no competing interests.

## Data Availability

All data including sampling regime, THS data, body size and ovarian metrics, as well as statistical analysis scripts are available on Scholarsphere (https://doi.org/10.26207/gqy8‐e215).

## Appendix

**TABLE A1:** All bees used in our cross‐species analysis.

Species	Type	Source	Q	W	M
bimac	col.4	Circleville Park	1		
bimac	col.2	Circleville Park		5	
bimac	col.10	Coyler Lake		5	2
bimac	col.11	Coyler Lake		5	
bimac	col.8	Tom Tudek Memorial Park	1		
bimac	Wild	Bear Meadows Natural Area		4	1
bimac	Wild	Benezette Elk Center	1		
bimac	Wild	Bernel Road Park		2	1
bimac	Wild	Circleville Park		1	
bimac	Wild	Coyler Lake		1	2
bimac	Wild	Penn State (UP)		2	4
bimac	Wild	Pine Grove Mills	1	1	2
bimac	Wild	Weiser State Forest		2	
gris	col.4	Tom Tudek Memorial Park		2	
gris	col.5	Tom Tudek Memorial Park	1	9	5
gris	col.6	Tom Tudek Memorial Park	1		
gris	col.7	Tom Tudek Memorial Park	1		
gris	Wild	Bear Meadows Natural Area			4
gris	Wild	Bernel Road Park		1	5
gris	Wild	Coyler Lake		1	1
gris	Wild	Rock Springs		3	
gris	Wild	Hyner View State Park		4	
gris	Wild	Penn State (UP)		1	
imp	col.5	Penn State (UP)	1		
imp	col.2	Penn State (UP)		16	
imp	col.9	Fairbrook Park		14	
imp	col.10	Hyner View State Park		12	5
imp	col^a^	wild		4	
imp	col.1	Commercial			5
imp	col.2	Commercial		5	
imp	col.3	Commercial		5	
imp	col.20	Commercial			
imp	col.22	Commercial	7	3	
imp	col.23	Commercial	10	5	
imp	col.25	Commercial		4	
imp	Wild	Bear Meadows Natural Area		5	6
imp	Wild	Circleville Park		1	
imp	Wild	Hyner View State Park		5	
imp	Wild	Penn State (UP)		6	5
imp	Wild	Tom Tudek Memorial Park		2	2
perp	col.4	Hyner View State Park	1		
perp	col.5	Scotia Pond	1		
perp	Wild	Bernel Road Park			2
perp	Wild	Circleville Park		2	1
perp	Wild	Coyler Lake			2
perp	Wild	Penn State (UP)		1	
perp	Wild	Pine Grove Mills	1	1	1
perp	Wild	Rock Springs			3
perp	Wild	Weiser State Forest			1
v/s	col.2	Black Moshannon State Park		5	
v/s	col.5	Hyner View State Park	1		
v/s	col.6	Hyner View State Park	1		
v/s	col^a^	Wild	1		1
v/s	Wild	Benezette Elk Center			1
v/s	Wild	Bernel Road Park		1	
v/s	Wild	Pine Grove Mills		1	2
v/s	Wild	Rock Springs		9	2
v/s	Wild	Weiser State Forest			3

*Note*: Type refers to whether the bee was captured from the wild or reared from a colony in the laboratory. Each colony number represents a unique colony for the associated species. Source indicates the site that bees were obtained from (in the case of wild bees used directly in our thermal assays and wild queens used for rearing) or whether they were purchased commercially. A separate THS analysis was conducted on just workers for these species (Figure 1b, Table 3). A separate analysis was also conducted on queens, workers, and males in *B. impatiens* to assess the effect of caste/sex on THS (Figure 1c, Table 3).

Abbreviations: bimac, *B. bimaculatus*; gris, *B. griseocollis*; imp, *B. impatiens*; M, male; perp, *B. perplexus*; Q, queen; v/s, *B. vagans*/*sandersoni*; W, worker.

^a^ The natal colony for these bees is unknown.

**TABLE A2:** Number of *Bombus impatiens* workers and queens assayed for each THS analysis.

Type	Source	Age‐Q				Age‐W		CO_2_ trials				RH1			RH2‐Q			RH2‐W			Col.	Sz.
		4	7	10	12+	7	14	2C	2T	7C	7T	20	50	70	20	70	90	20	70	90		
Wild	BM																					4
Wild	CV																					1
Wild	HV																					4
Wild	PS																					6
Wild	TP																					2
col.5	Wild																1					
col.2	Wild					7	3													16	16	16
col.9	Wild					5	4													14	14	13
col.10	Wild						2					3	3	3						12	12	12
col.^a^	Wild						4													4	4	4
col.1	Comm											3	3	3								
col.2	Comm											3	3	3						5	5	5
col.3	Comm											3	3	3						5	5	5
col.20	Comm																	10	10			
col.22	Comm	4		3	7			3	4	4	4						7			3	3	
col.23	Comm	5	4	6	10			5	4	5	5				10	10	10			5	5	
col.25	Comm	8																		4	4	

Abbreviations: Age‐Q, queen age trials; Age‐W, worker age trials; CO_2_ Trials, CO_2_ narcosis, ovary activation, and presence of brood analyses for queens; Col., colony effect analysis among laboratory‐reared workers; RH1, initial humidity trial with air incubators with workers; RH2‐Q, second humidity trial with queens; RH2‐W, second humidity trial with workers; Sz., body size analysis among wild and laboratory‐reared workers.

^a^ The natal colony for these bees is unknown.

*Source*: BM, Bear Meadows Natural Area; CV, Circleville Park; HV, Hyner View State Park; PS, Penn State–University Park Campus; TP, Tom Tudek Memorial Park.
